# Long-term RNA persistence in postmortem contexts

**DOI:** 10.1186/2041-2223-4-7

**Published:** 2013-04-23

**Authors:** Sarah L Fordyce, Marie-Louise Kampmann, Nienke L van Doorn, M Thomas P Gilbert

**Affiliations:** 1 Centre for GeoGenetics, Natural History Museum of Denmark, Øster Voldgade 5-7, 1350, Copenhagen K, Denmark; 2 BioArCh, Department of Archaeology, Biology and Chemistry, S-Block, University of York, York, YO10 5YW, UK

**Keywords:** Ribonucleic acid (RNA), Postmortem, Forensic, Paleogenetics, Instability

## Abstract

Ribonucleic acids (RNA) are generally considered fragile molecules that are readily degraded. However, there is growing documentation of long-term (from days to centuries) RNA persistence in a variety of contexts and tissue types, and as such a number of academic disciplines are beginning to exploit degraded RNA. While the reasons for its survival are not fully understood, there are several plausible mechanisms that would safeguard this molecule against degradation. However, after examining the literature available on the postmortem instability and decay mechanisms of RNA, it has become clear that limited experimental studies and no reviews offer an overview of these mechanisms. Hence in this review we outline molecular reasons for RNA surviving long-term postmortem, and provide specific examples of RNA survival in forensic, archival and archaeological contexts. A better understanding of the mechanisms of RNA decay will be crucial for developing expectations on its long-term survival.

## Review

### Introduction

The potential of RNA as a source of genetic information remains relatively unexplored in postmortem studies. This is possibly due to the well-documented phenomena that in many common situations, RNA degrades more readily than DNA. However, RNA can provide us with valuable information not directly evident in the genome and hence could be worth examining further in postmortem samples. For instance, unlike the genome, RNA transcripts provide access to gene regulation and protein information [1]. Moreover, contrary to the genome, the transcriptome reflects the genes that are actively expressed at any given time and can vary with external conditions.

Mechanisms behind DNA degradation and its effects on ancient DNA (aDNA) were reviewed by Lindahl [2] in a paper that has aided understanding of when and where one could expect DNA to survive based on the patterns of degradation. Similarly, to be able to scrutinize studies reporting the long-term survival of RNA, one must ask the question: is it likely that RNA would survive under these conditions? Understanding the mechanisms of RNA instability and decay will aid in the interpretation of reports of RNA survival.

### RNA structure and degradation mechanisms

The half-life of nucleic acids is limited by several endogenous (for example, structure, nature of the bases, sugars and phosphate residues) and exogenous factors (for example, pH, presence of metal cations, ultraviolet light, presence of oxygen and water [2,3]). Moreover, factors influencing the rate of RNA degradation are unique in different postmortem scenarios. For instance, RNA degradation in a deceased individual or body parts occurs predominantly due to the enzymatic activity of cellular RNases. On the other hand, in dried biological materials, such as blood or saliva stains or mummified tissue, the samples are dehydrated. In dehydrated conditions, RNase activity is significantly reduced, therefore, in this scenario RNA degradation occurs mostly due to physical and chemical factors.

While DNA fragments of quality that would enable conventional PCR analyses are estimated to survive at least 100,000 years at the colder extremes of the ambient temperatures naturally found on Earth [4], conventional wisdom based on common lab experience of rapid RNA degradation would suggest that survival of similar quality RNA might be significantly less. However, to consider whether this is really so, and to understand what such degradation may encompass, it is helpful to consider RNA structure and how it differs to DNA.

**Figure 1:**
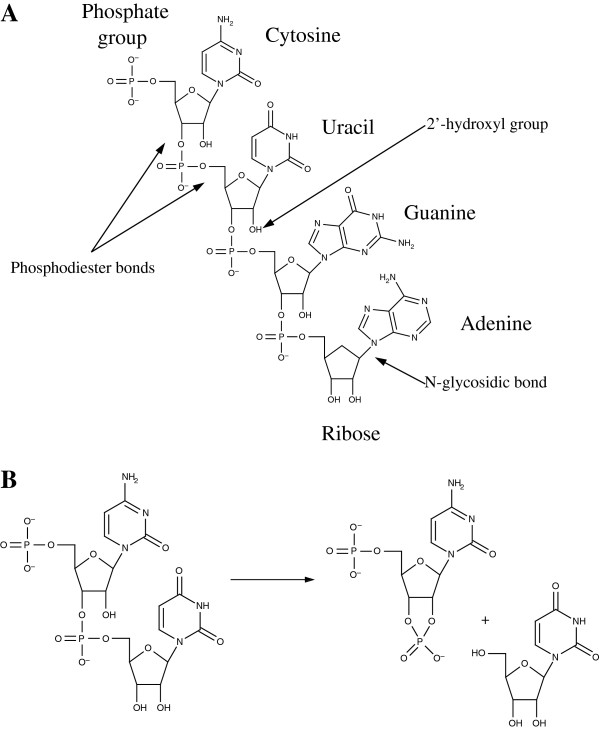
**Structure of RNA and process of hydrolysis.** (**A**) Chemical structure of RNA. The ribose, bases and phosphate group are labeled. The 2′-hydroxyl group (labeled) allows the RNA molecule to be more easily degraded via hydrolysis than DNA. Similarly, the phosphodiester bond (also labeled) in RNA can be broken during hydrolysis. The N-glycosidic bond (labeled) is stronger in RNA than DNA. (**B**) The chemical process of hydrolysis, where the 2′-hydroxyl group has attacked the adjacent phosphodiester bond, cleaving the backbone of the RNA.

In contrast to DNA, the RNA molecule contains a hydroxyl group (2′-OH) at the 2′ position of the sugar (see Figure 1A), and it is this group that is one of the greatest causes of its structural instability, as, when present in flexible regions of the molecule, it has the ability to chemically attack the adjacent phosphodiester bond (Figure 1A) and thus cleave the backbone [5] (Figure 1B). Indeed, in this regard Lindahl [6] has argued that the phosphodiester bonds in a DNA chain are 200 times more stable than those in an RNA chain at neutral pH when in the presence of physiological concentrations of Mg^2+^.

Several conditions make RNA more susceptible to hydrolytic damage than DNA. For instance, the susceptibility of RNA to hydrolysis is increased in the presence of cations, such as Ca^2+^, and transitional metals [7]. Additionally, alkali conditions, including the levels naturally found inside cells, increase the susceptibility of RNA to hydrolysis [5]. The implication that RNA rapidly hydrolyzes in the presence of these cations, transitional metals and alkali conditions, is based on the ubiquity of these substances and the fact they are essential to all known living organisms. Archaeological and forensic specimens are likely to come into contact with these substances, thus increasing the possibility for RNA degradation.

Conversely, while RNA is susceptible to hydrolysis, there are some instances where it is more stable than DNA. Depurination and depyrimidation are the processes in which alterations to DNA occur, causing purine and pyrimidine bases, respectively, to be removed from the sugar by hydrolysis of the beta-N-glycosyl bond between them. This results in the replacement of purine or pyrimidine with a hydroxyl group. However, while RNA has weaker phosphodiester links, it has stronger N-glycosidic bonds (see Figure 1A), and thus the rates of depurination of RNA are considerably reduced in comparison to those in DNA [8]. For instance, Kothcekov and Budowsky [9] estimate that these processes occur 100 to 1,000 times slower in RNA than DNA.

RNA’s ability to withstand depurination and depyrimidation processes, more so than DNA, could be significant for the long-term survival of RNA in postmortem conditions. The reasoning for this is that DNA fragmentation caused by hydrolytic depurination is assumed to be the fastest of spontaneous chemical reactions limiting the half-life of amplifiable aDNA [2,10], resulting in a loss of base information and difficulties with *in vitro* replication of the damaged molecule by PCR [11]. Hence, if these processes are reduced in RNA, perhaps it is not surprising that this molecule could persist in certain postmortem tissues.

A further relevant feature is that RNA readily forms secondary and tertiary structures, and these can have a distinct effect on the rate and specificity of RNA phosphodiester bond hydrolysis. In particular, ribosomal RNA (rRNA) is more stable than messenger RNA (mRNA), most likely due to the ability of rRNA to be partially double-stranded and form secondary structures. The difference in RNA degradation rates of these two RNA species forms the basis of postmortem interval determination (see Forensic potential of RNA). Tertiary structures are further stabilized by ionic interactions, for instance Mg^2+^ stabilizes the native tertiary structure of transfer RNA (tRNA) [7]. Hence, it is likely that these secondary and tertiary structures are partially responsible for reducing the effects of degradation in postmortem RNA.

Additionally, micro RNA (miRNA) is exceptionally stable postmortem, which may be attributed to its ability to bind to proteins and subcellular compartmentalization [12]. Moreover, the latter point may also be a general mechanism of RNA protection, which has been described for cell free RNA in plasma and saliva [13].

Although RNA in itself is a fragile molecule, there are several factors safeguarding it, and in some situations it may be expected to persist, thus opening up possibilities for exploitation in a range of fields. So far this has encompassed the forensic, medical and archaeological sciences.

### Examples of long-term RNA persistence

#### Forensic potential of RNA

The potential utility of RNA for the field of forensic science is a recent concept [14,15]. The reason for this is most likely that analyzed RNA sequences would not normally be expected to hold information that could be used to identify an individual. RNA has, however, been suggested as a potentially informative tool with regards to determining the cellular origin of a sample and for generating an estimate of the time of sample deposition [16]. Moreover, RNA can be co-extracted with DNA to provide additional information to routine DNA analyses [17,18].

Postmortem RNA degradation has been explored in a variety of tissue types. For example, Marchuk *et al*. [19] indicated that RNA remains unaffected for up to 96 hours postmortem in brain tissue, cartilage, tendon ligament and lung tissue. Inoue *et al*. [20] also reported the long-term stability of rRNA in the liver for two days and in the brain for up to seven days. Dehydrated adipose tissue [21] has significant prolongation of RNA integrity, probably because the lack of water reduces the activity of RNase. Similarly, RNA from blood samples has been detected in samples up to sixteen years old [22,23]. These authors found that dried bloodstains or dehydrated samples contained reduced RNase activity and overall RNA degradation due to the reduction of water [22,23]. Additionally, total RNA and mRNA from trabecular bone [24] and bone marrow [25] have been detected up to five days postmortem, using reverse transcriptase PCR (RT-PCR). Finally, King *et al*. [26] report hair root as a useful source of mRNA for genetic tests, with mRNA being detected after ten days of storage at room temperature after plucking.

Although mRNA has been demonstrated to survive for extended periods of time, mRNA is nonetheless highly susceptible to decay, with the half-life of various mRNA molecules varying between several weeks, hours, or even minutes for specific induced genes [27]. This differential rate of mRNA degradation provides us with the possibility to predict the time since death interval and wound age determination by assessing RNA integrity [28-30]. Bauer *et al*. [22,31], for example, were able to detect significant differences in RNA degradation levels by examining mRNA integrity in dried blood, reporting a significant correlation between the age of bloodstains and mRNA degradation up to 4 to 5 years.

As an intermediate for protein synthesis, mRNA levels reflect tissue-specific gene expression, as different tissues require different levels of proteins for specific enhanced expression [32]. This has lead to the use of RNA in forensics to identify tissues and body fluids, such as saliva, semen, venous blood and menstrual blood [23,33-37]. Furthermore, miRNA markers have been studied for potential use in body fluid determination [33,38]. The obvious advantage of miRNAs over mRNAs is their small size (typically 18 to 22 nucleotides), making their retrieval more likely from degraded samples, particularly in a postmortem context. For instance, just as mRNAs have been used to identify sources of bodily fluids, one study applied genome-wide microarrays covering more than 700 miRNAs to samples from all body fluids relevant to forensic studies [38].

It should also be noted that different mechanisms of degradation (that is, in dehydrated or fresh samples) suggest that different features of RNA species would determine their postmortem survival. For instance, it has been established that the RNA decay rate within living cells is associated with the presence of AU-rich motifs in the RNA sequence [39]. Catts *et al*. [39] demonstrated that these motifs are crucial for degradation in postmortem brain tissue. Hence, the selection of RNA markers for medical applications should consider this factor. Conversely, such sequence motifs are unlikely to influence RNA degradation in dried samples (for example, blood and saliva), a typical subject in forensic studies.

The examples above drawn from forensic sciences highlight that informative amounts of RNA can clearly survive postmortem, conditional on tissue type and the conditions at death/burial. It should, however, be emphasized that the majority of forensic RNA investigations have been carried out in controlled conditions with samples that have been kept in known storage conditions. Exposure to sunlight, humidity, high temperature and other unfavorable influences might either lead to total loss of extractable RNA or RNA fragmentation might be accelerated. Hence, based on the success of the age determination and sample type, and the extended persistence of RNA in various tissue types (dried blood being the most favorable), additional investigation into the long-term survival of RNA in both a laboratory setting and in genuine casework conditions is warranted.

#### RNA persistence in archival material

Formalin-fixed paraffin-embedded (FFPE) tissue samples are the most common form of pathologic tissue collection [40] and offer a source of pathologically disease-specific material for potential use in molecular investigations of both DNA and RNA [41,42]. FFPE material can also be used to detect human transcriptomes for cancer genetic studies [43]. Although the recovery of RNA from FFPE specimens is challenging (reviewed in detail in [44]), when stored appropriately, RNA has been detected for at least 40 years after fixation [41,45].

Improved extraction and amplification techniques have resulted in a proliferation of cancer-focused gene-expression studies using RNA from FFPE tissue, as previously shown [46,47]. Whole transcriptome amplification has been shown to relatively accurately maintain differences in relative gene abundance comparing FFPE tumor and benign tissue [47] in FFPE benign and malignant glands, reporting a clear difference in the expression profiles between the malignant and benign samples, a result that was further supported by Dunn *et al*. [48]. Ravo *et al*. [49] reported similar reproducible gene expression profiles in FFPE breast cancer samples that were 6 to 19 years old. To date, the vast majority of FFPE cancer gene expression studies have focused on validating FFPE RNA extraction and gene expression methods. However, given the current success of the validation studies, it seems likely that FFPE cancer gene expression studies will provide a valuable clinical tool for detecting and diagnosing cancer and other disease.

FFPE archival material has also provided a means to retrospectively study infectious disease, providing a means of clarifying pathogen evolution and emergence. For example, two historically important pathogens have been studied through use of FFPE archival material, namely 1918 Spanish influenza and human immunodeficiency 1 virus (HIV-1) [41,42].

Genomic RNA from the 1918 influenza virus was recovered from archived formalin-fixed lung autopsy materials and from frozen, unfixed lung tissues from an Alaskan influenza victim who was buried in permafrost in November 1918 [41,50,51]. RNA from this archival material allowed the complete coding sequences of all eight viral RNA segments to be determined, allowing insight into the nature and origin of this pathogen. The resulting RNA sequences were used to generate an influenza virus, using reverse genetics, containing all eight gene segments of the pandemic virus to study the properties associated with its extreme virulence [52]. The authors present sequence and phylogenetic analyses (although these analyses have since been disputed as in [53]) to propose that the 1918 virus was not a reassortment virus, but more likely to be of avian origin, much like the current H5N1 virus. This analysis could provide insight into the pathogenicity and virulence of currently circulating and novel influenza viruses.

Similarly, Worobey *et al*. [42] reported the partial sequence of an HIV-1 group M virus recovered from an FFPE archival sample originating from Kinshasa (Democratic Republic of Congo) that dated to 1960. Although a single sample, the fact it was nearly 20 years older than almost all other known HIV-1 viral sequences enabled the authors to both obtain a snapshot of the diversity of the HIV-1 epidemic at this date, and better calibrate molecular clock analysis of the last common ancestor of HIV-1 M in humans. In this regard, the authors report an estimated start of the epidemic c.1900, almost 30 years prior to other estimates. Although this may at first not sound significant, in the context of colonial African history and thus possible causes of the start of the epidemic, this is a considerable difference, with the authors suggesting that the start of the epidemic was directly linked with the foundation and initial growth of Central/West African colonial cities as opposed to previous hypotheses linking it with the increased mobility of populations in later decades.

#### Archaeological potential of RNA

In addition to providing insights into recent sample types such as forensic and medical samples, RNA has potential to contribute to the field of ancient biomolecules [54]. It is increasingly being appreciated that phenotypic differences between organisms arise not due to mutations that change the sequence of protein-coding genes, but to changes to the activities of the genes [55]. In this regard, the characterization of RNA may be of particular interest to scenarios where rapid intra-specific phenotypic change is observed, for example, in domestication studies. However, the study of RNA from archaeological material remains a relatively unexplored field. Extracting and sequencing RNA in preserved materials presents challenges, since the nucleic acids are often neither pure nor intact.

The primary tissue type that has received attention in the field of archaeological RNA is desiccated seeds. It has traditionally been thought that after prolonged storage, a seed loses its capacity to germinate. However, with the germination of a 2,000-year-old date palm and, more recently, the germination of a 30,000-year-old *Silene stenophylla* seed, it has become apparent that this is not the case [56,57]. If the seed is kept in a dry environment, it undergoes a process of spontaneous mummification, and the external morphology is preserved, with only slight discoloration occurring [58].

Cheah and Osborne [59] also demonstrated that some very low molecular weight nucleic acids were found in Neolithic grains from Egyptian tombs. In 1985, Rollo [58] analyzed ancient cress (*Lepidium sativum* L.) seeds found in the Thebes Necropolis, dated approximately 1,400 years BC. These seeds were found to contain low molecular weight (10 bp) fragments of RNA, based on the detection of uracil using mass spectrometry analysis. Hybridization tests were performed to confirm that the RNA was of plant origin and not bacterial DNA. However, the possibility of modern contamination was not excluded. In 1990, Venanzi and Rollo [60] claimed to have found RNA in ancient maize and cress seeds (1,000 and 3,000 years old) and went on to suggest that the nucleic acids in the ancient samples were composed mainly of RNA. Following this study, analysis of ancient RNA from seeds halted, perhaps because the RNA fragments reported were not long enough to be useful for any conventional RT-PCR based sequence analyses. Another significant reason RNA work ceased was likely to be because of the inability to authenticate the source of the RNA found in the ancient seed samples.

However, using second-generation sequencing, a recent study [61] has successfully sequenced genuinely ancient RNA derived from ancient (725 year-old) desiccated maize seeds. This study indicates that similar to the report of Venanzi and Rollo [60], RNA was better preserved than DNA, based on fragment length analyses. Furthermore, the study demonstrates the ability to obtain mRNA sequences of informative length, resulting in gene identification. Hence, this study indicates that due to advances in sequencing techniques [62], ancient RNA (paleotranscriptomic) studies may provide important insights into crop domestication, and potentially other archaeological studies.

## Conclusions

Although the fate of RNA *in vivo* is to be degraded, the extent of its survival postmortem is perhaps more than many would expect. The examples highlighted above not only provide evidence for RNA being more robust than previously considered, but also demonstrate the tissue types and environmental conditions in which one could expect RNA persistence. A common theme seen in all of the sample types (forensic, archaeological and archival) is that in order for RNA to be preserved, there needs to be some form of barrier or macromolecular structure between the environment and the nucleic acids. This is true for archaeological specimens (such as seed casing and virions) and for archival material (where the FFPE acts as the barrier). These barriers protect RNA from the two main sources of degradation, oxygen and water, as well as other environmental factors.

In conclusion, RNA can provide valuable information that is otherwise unobtainable through DNA analysis. As such, the long-term survival of RNA warrants further exploration and characterization in future studies.

## Abbreviations

Bp: Base pairs; FFPE: Formalin-fixed paraffin-embedded; ADNA: Ancient DNA; PCR: Polymerase chain reaction; 2′-OH: 2 prime hydroxyl group; RT-PCR: Reverse transcriptase polymerase chain reaction; MRNA: Messenger RNA; MiRNA: MicroRNA; RRNA: Ribosomal RNA; TRNA: Transfer RNA.

## Competing interests

The authors declare no competing interests.

## Authors’ contributions

SLF, MLK, NLVD and MTPG participated in the writing of the manuscript. All authors read and approved the final manuscript.
